# Supplementary material for: Detailed molecular and epigenetic characterization of the pig IPEC-J2 and chicken SL-29 cell lines
Source: iScience. 2023 Feb 20;26(3):106252. doi: 10.1016/j.isci.2023.106252 (PMC10018572; doi:10.1016/j.isci.2023.106252)
Supplement: Data S2. Complete homer output for identified motifs in Chicken SL-29, related to Tables 5 and 6 — Homer motif analysis results for histone modifications H3K4me1, H3K4me3, H3K27ac, enhancers, and ATAC-seq of chicken SL-29 cell line. Parameters for possible false positives is as mentioned earlier for S5. [file mmc3.zip › Data_S2/S6/Chicken_SL_29/peak_files_H3K27ac/homerResults/motif1.similar.html]

motif1

## Information for motif1

A
C
T
G
G
T
C
A
A
C
T
G
A
C
T
G
A
G
T
C
A
G
T
C
A
G
C
T
C
G
T
A
  
Reverse Opposite:  

G
C
A
T
C
T
G
A
A
C
T
G
A
C
T
G
A
G
T
C
A
G
T
C
A
C
G
T
A
G
T
C
  

|  |  |
| --- | --- |
| p-value: | 1e-86 |
| log p-value: | -1.987e+02 |
| Information Content per bp: | 1.909 |
| Number of Target Sequences with motif | 2828.0 |
| Percentage of Target Sequences with motif | 9.38% |
| Number of Background Sequences with motif | 1938.2 |
| Percentage of Background Sequences with motif | 6.41% |
| Average Position of motif in Targets | 150.5 +/- 82.4bp |
| Average Position of motif in Background | 148.3 +/- 91.9bp |
| Strand Bias (log2 ratio + to - strand density) | 0.1 |
| Multiplicity (# of sites on avg that occur together) | 1.12 |
| Motif File: | file (matrix) reverse opposite |

### Similar de novo motifs found

|  |  |  |  |  |  |  |  |
| --- | --- | --- | --- | --- | --- | --- | --- |
| Rank | Match Score | Redundant Motif | P-value | log P-value | % of Targets | % of Background | Motif file |
| 1 | 0.720 | T C G A C T A G C T A G A T G C A G T C A T G C A T G C T A C G | 1e-68 | -157.890516 | 18.46% | 14.76% | motif file (matrix) |
| 2 | 0.768 | T A C G T A C G T A G C A T G C T C A G C T A G A T C G A G T C A G T C A G C T | 1e-68 | -157.791191 | 16.13% | 12.64% | motif file (matrix) |
| 3 | 0.733 | C T G A A T C G T A C G T A G C A G T C A C T G A G T C A C G T A C G T A G C T | 1e-61 | -140.889735 | 40.81% | 36.19% | motif file (matrix) |
| 4 | 0.620 | C T G A A C T G T G A C T C G A T A C G T A G C A T G C G C A T | 1e-53 | -122.373633 | 25.62% | 21.88% | motif file (matrix) |
| 5 | 0.645 | T G C A C T A G A C T G A T G C A G T C A G T C A C G T G T C A | 1e-49 | -113.085698 | 42.51% | 38.34% | motif file (matrix) |
| 6 | 0.606 | T A C G T A G C T A C G T A C G A T G C A T G C G A T C C A G T G T C A C T G A | 1e-48 | -111.471864 | 32.34% | 28.48% | motif file (matrix) |
| 7 | 0.640 | C G T A A C T G A C T G A G T C A G T C A C T G A G T C A C T G A C T G A C T G A G T C A G T C A G T C | 1e-32 | -73.973755 | 4.71% | 3.40% | motif file (matrix) |
| 8 | 0.617 | A C G T A G T C A C G T A T C G A C T G A C T G A G T C A G T C | 1e-29 | -68.664812 | 14.75% | 12.52% | motif file (matrix) |
| 9 | 0.763 | C T A G A C G T C G T A T A C G A C T G G T A C A G T C A C T G A G T C G T C A | 1e-10 | -24.089673 | 0.15% | 0.05% | motif file (matrix) |
